# Delimiting species, revealing cryptic diversity, and population divergence in Qinghai‐Tibet Plateau weevils through DNA barcoding

**DOI:** 10.1002/ece3.11592

**Published:** 2024-07-08

**Authors:** Jinliang Ren, Li Ren, Runzhi Zhang

**Affiliations:** ^1^ Key Laboratory of Zoological Systematics and Evolution, Institute of Zoology Chinese Academy of Sciences Beijing China; ^2^ College of Life Science University of Chinese Academy of Sciences Beijing China

**Keywords:** biodiversity, cryptic species, DNA barcode, species delimitation, taxonomy

## Abstract

The *Leptomias* group represents one of the most diverse taxonomic group of weevils in the Qinghai‐Tibet Plateau and its adjacent areas. Despite the potential of hidden diversity, relatively few comprehensive studies have been conducted on species diversity in this taxonomic group. In this study, we performed DNA barcoding analysis for species of the *Leptomias* group using a comprehensive DNA barcode dataset that included 476 sequences representing 54 morphospecies. Within the dataset, our laboratory contributed 474 sequences, and 390 sequences were newly generated for this study. The average Kimura 2‐parameter distances among morphospecies and genera were 0.76% and 19.15%, respectively. In 94.4% of the species, the minimum interspecific distances exceeded the maximum intraspecific distances, indicating the presence of barcode gaps in most species of *Leptomias* group. The application of Automatic Barcode Gap Discovery, Assemble Species by Automatic Partitioning, Barcode Index Number, Bayesian Poisson tree processes, jMOTU, and Neighbor‐joining tree methods revealed 45, 45, 63, 54, and 55 distinct clusters representing single species, respectively. Additionally, a total of four morphospecies, *Leptomias kangmarensis*, *L. midlineatus*, *L. siahus*, and *L.* sp.9RL, were found to be assigned to multiple subclade each, indicating the geographical divergences and the presence of cryptic diversity. Our findings of this study demonstrate that Qinghai‐Tibet Plateau exhibits a higher species diversity of the *Leptomias* group, and it is imperative to investigate cryptic species within certain morphospecies using integrative taxonomic approaches in future studies. Moreover, the construction of a DNA barcode reference library presented herein establishes a robust foundational dataset to support forthcoming research on weevil taxonomy, phylogenetics, ecology, and evolution.

## Graphical abstract

The *Leptomias huangi* Chao, 1981 mating on *Bidens* sp. (Asterales: Asteraceae).

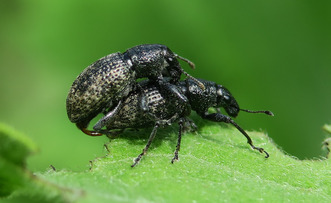

## 1 INTRODUCTION

The Qinghai‐Tibet Plateau (QTP), boasting the largest and highest expanse of its kind globally, exhibits an average elevation surpassing 4000 m (Zhang et al., 2002). Due to their exceptional species richness and endemism, the Himalayas and Hengduan mountains have been recognized as one among the 25 biodiversity hotspots worldwide (Myers et al., 2000). Previous research has demonstrated that intricate geographical environments can result in species differentiation and speciation among plants (Favre et al., 2016; Zhang et al., 2014), birds (Lei et al., 2014; Liu et al., 2016), and insects (Huang et al., 2008; Li et al., 2020). Despite a significant constituent of the insect community in the QTP, no comprehensive investigation into the concealed diversity and population divergence has been conducted for the *Leptomias* group.

**FIGURE 1:**
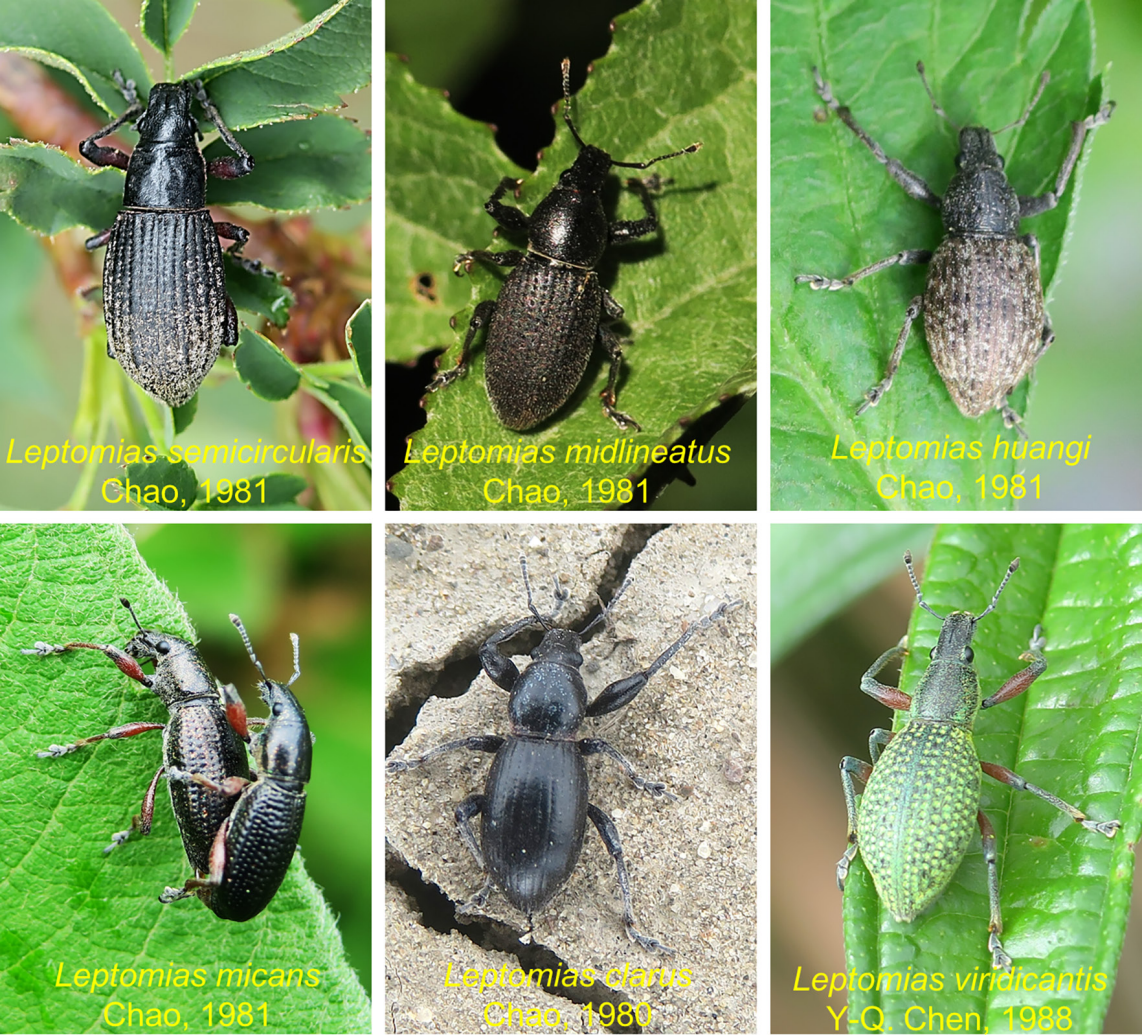
Some species of *Leptomias* group.

The *Leptomias* group (Coleoptera: Curculionoidae: Entiminae) comprises seven genera, namely *Geotragus*, *Pachynotus*, *Leptomias*, *Hyperomias*, *Xizanomias*, *Triangulomias*, and *Odontomias* (Chao & Chen, 1980; Chen, 1991). This group is distributed worldwide with the majority diversity in the QTP and its adjacent areas (Alonso‐Zarazaga et al., 2023). Species of this group have a wide range of hosts, and most of them harm the main agricultural crops and forestry in the QTP region. For example, *Leptomias longisetosus* and *L. crassus* damage wheat and barley, and *L. semiacularis* also poses a threat to apple trees, poplars, and willows, with the exception of barley and rape crops (Wang et al., 1993). The identification and classification of species of the *Leptomias* group have faced significant challenges due to the large number of species, morphological similarities among counterparts (Figure 1), the interference caused by different life stages (Ma et al., 2022), and missing taxonomic expertise (Rosas et al., 2011). Therefore, it is necessary to systematically and extensively evaluate the diversity of the *Leptomias* group based on the DNA‐based approaches, which is of great value for the species identification and pest management of this group.

Tautz et al. (2002) advocate a classification of species based on DNA sequence data (DNA taxonomy). DNA barcoding, a method for species identification based on standard gene markers, for example, the mitochondrial cytochrome oxidase subunit I (COI) gene for animals, has become a reliable basis for assembling reference sequence libraries of known species and discovering new species in many taxonomic groups (Hebert et al., 2003; Hebert et al., 2004). The efficacy of this approach has been successfully demonstrated across various taxa of Coleoptera, including Curculionidae (Astrin et al., 2012; Ma et al., 2022; Yang et al., 2021), Coccinellidae (Huang et al., 2020), and Tenebrionidae (Li et al., 2020). Previous studies have also shown the effectiveness of DNA barcoding in species identification and diagnosis within the Curculionoidea (Germann et al., 2017; Ma et al., 2022). In addition to the identification of known species, COI barcodes have also been proven to be suitable for elucidating cryptic species (Craft et al., 2010; Janzen et al., 2017; Velona et al., 2015), unveiling biogeographic patterns (Chen et al., 2018; Dinca et al., 2021), exploring population genetics (Gomez et al., 2018; Lin et al., 2020), and reconstructing phylogenetic relationships (Cognato et al., 2020; Grebennikov, 2022; Hajibabaei et al., 2007) at the species level. In the field of ecology, COI barcodes are utilized for analyzing organisms' feeding habits, conducting environmental monitoring (Valentini et al., 2009), identifying intestinal microorganisms (Bucklin et al., 2011), detecting invasive species (Sukirno et al., 2020), and exploring biodiversity (Chimeno et al., 2022; Leray & Knowlton, 2015; Smith et al., 2008).

Analytical approaches for DNA barcode data analysis play a crucial role in species delimitation and biodiversity assessments (Ge et al., 2021). However, the use of different approaches may yield various species boundaries and generate distinct putative species. For example, the Automatic Barcode Gap Discovery (ABGD) algorithm identifies a divergence gap that corresponds to the differentiation between intraspecific and interspecific distances. The effectiveness of this method in species delimitation is limited without the inclusion of such a gap (Puillandre et al., 2012; Song et al., 2018). Assemble Species by Automatic Partitioning (ASAP), proposed by Puillandre et al. (2021), is a new method to build species partitions from single locus sequence alignments. Furthermore, the widely utilized Barcode of Life Data System (BOLD) and Barcode Index Number (BIN) system employ diverse distance metrics to construct a Neighbor‐joining (NJ) tree, subsequently establishing a persistent registry for life OUT in BOLD (www.bold.system.org; Ratnasingham & Hebert, 2013). And the Bayesian Poisson tree processes (bPTP) model considers the number of substitutions between branching and speciation as independent events (Zhang et al., 2013). Additionally, jMOTU can efficiently and robustly identify molecular classification groups present in survey datasets within a short timeframe (Jones et al., 2011). And finally, Neighbor‐joining (NJ) tree considers the phylogenetic signal of the sequences and attains higher classification accuracy (Saitou & Nei, 1987).

In the present study, we generated a DNA barcode dataset comprising 476 sequences (474 of which were contributed by our laboratory, and 390 sequences were newly generated in the current study) through sample collection and molecular experiments conducted over the past 5 years. Subsequently, comprehensive analyses encompassing genetic distances, species delimitation, and population structures were performed to investigate the hidden species diversity and population divergence species of the *Leptomias* group in the QTP.

## 2 MATERIALS AND METHODS

### 2.1 Sample collection and identification

Fieldworks were conducted in the Xizang autonomous region of China from 2018 to 2022 (Table S1). The longitude, latitude, and altitude of the specimens were initially recorded. Subsequently, the specimens were preserved in 95% ethanol at −20°C until further analysis. All collected specimens underwent morphological identification by experts. To enhance the representativeness of our study, we aimed to collect specimens from various locations across the QTP, ensuring maximum coverage and facilitating comprehensive observations of intraspecific variation. Detailed information for each specimen, including sampling locality, BOLD process ID, and GenBank accession number, was documented in Table S1.

### 2.2 DNA extraction, amplification, and sequencing

We utilized DNeasy Blood & Tissue Kits (QIAGEN, Germany) to extract DNA from all specimens. Depending on the size of each specimen, DNA was extracted from either 1, 3, 6 legs or from the entire body (Ma et al., 2022). All voucher specimens were preserved at the Institute of Zoology, Chinese Academy of Sciences. Polymerase chain reaction (PCR) amplifications for COI sequences were performed using the primers LCO1490 (GGTCAACAAATCATAAAGATATTGG) and HCO2198 (TAAACTTCAGGGTGACCAAAAAATCA) (Folmer et al., 1994). PCR reaction mixes (25 mL) contained 12.5 μL 2× Taq PCR MasterMix (Tiangen Biotech Co., Ltd., Beijing, China), 1 μL of forward and reverse primer each (Sangon Biotech Co. Ltd., Shanghai, China), 2 μL total undiluted DNA template, and 8.5 μL dd H_2_O. PCR profile as follows: 94°C for 2 min, first cycle set (five repeats): 94°C for 40 s, 45°C for 40 s, and 72°C for 60 s. Second cycle set (35 repeats): 94°C for 40 s, 51°C for 40 s, and 72°C for 60 s, followed by elongation at 75°C for 5 min. The PCR products were visualized through 1% agarose gel electrophoresis in TAE buffer. Subsequently, the successful PCR products were sent to the Beijing Genomics Institute (BGI, Shenzhen, China) for sequencing. The obtained raw data were then assembled and edited using SeqMan software (ver. 7.1) (Burland, 2000). Subsequently, all sequences were subjected to translation into amino acids using MEGA ver. 7 in order to verify and prevent the occurrence of stop codons. Finally, the sequences generated from this study have been deposited in both the GenBank and Barcode of Life Data System (BOLD) (Table S1).

### 2.3 Data integration, genetic distance, and phylogenetic analysis

We have also retrieved all COI barcode sequences of species of *Leptomias* group previously published from BOLD and GenBank databases. After excluding sequences with less than 600 bp and those containing degenerate or missing bases, we were able to obtain only two sequences from the public database (Table S1).

We utilized MEGA 7.0 to record the nucleotide composition, conserved sites, variable sites, parsimony‐information sites, and singleton sites (Kumar et al., 2016). Additionally, we employed the Kimura 2‐parameter (K2P) model to calculate intraspecific genetic distances (intra‐GD) and interspecific genetic distances (inter‐GD) (Kimura, 1980). We utilized Origin 2018 to visualize and represent the distribution of genetic distances through histograms and scatter plots (Moberly et al., 2018). Identical sequences were condensed into unique haplotypes using DNAsp, which were subsequently employed for phylogenetic analyses (Li et al., 2023; Librado & Rozas, 2009). Phylogenetic inference analyses were conducted using maximum likelihood (ML). Optimal nucleotide substitution models and ML analyses were selected based on the Akaike information criterion through the IQ‐TREE web server (Trifinopoulos et al., 2016). The ML analyses were performed with the following settings: ML + rapid bootstrap (Hoang et al., 2018), 1000 replicates, and the GTR + F + I + G4 model. Haplotype networks for some species complexes were constructed using TCS in POPART (Clement et al., 2000; Leigh & Bryant, 2015).

### 2.4 Species delimitation

The six species delimitation methods, namely ABGD, ASAP, jMOTU, BIN, bPTP, and NJ, were used to evaluate the species boundaries and identify potential molecular operational taxonomic units (MOTUs). The ABGD analyses were performed at the web server (http://wwwabi.snv.jussieu.fr/public/abgd/abgdweb.html) (Puillandre et al., 2012). The following configuration was utilized: A relative gap width of *X* = 1.0, K2P distance metric, intraspecific divergence (*p*) values ranging from .001 to .1, while other parameters were remained at their default settings. The ASAP analyses were performed at the web server (https://bioinfo.mnhn.fr/abi/public/asap/asapweb.html) (Puillandre et al., 2021), K2P distance metric, choose the result with the lowest score. The jMOTU analyses were performed at the analytical package with parameters to use for MOTU definition 1 to 50, a low BLAST identity filter at 97%, percentage of minimum sequence length at 95%, and number of processors to use in Magablast at 4 (Jones et al., 2011). The BIN, a widely employed feature at BOLD system, was directly queried and tallied during analysis (up to 2023.7.10). The bPTP analyses were performed on the web server (http://species.h‐its.org/ptp/; Zhang et al., 2013) using the following setting: 500,000 MCMC generations, with the initial 20% of trees discarded as burn‐in. Analysis of the NJ trees was performed in MEGA 7.0 using Gap/Missing Data Processing parameters of “Paired Deletion” (Kumar et al., 2016). When four or more species delimitation methods grouped sequences of morphospecies into a single MOTU, we considered the results of both morphological identification and molecular species delimitation to be consistent. Conversely, if sequences of the same morphospecies were grouped into more than one MOTUs by four or more species delimitation methods, or if sequences of different morphospecies were grouped into the same MOTU, we classified these cases as “taxonomic warnings” (Li et al., 2023).

## 3 RESULTS

### 3.1 DNA barcode dataset of *Leptomias* group

We successfully constructed a comprehensive DNA barcode dataset consisting of 476 sequences representing 54 morphospecies from seven genera in QTP (Table S1). The dataset yielded a total of 180 unique haplotypes (Table S2). It was observed that among the species analyzed, 21 species had only two sequences, while more than two sequences were obtained for 32 species. Only one species had a single sequence. Notably, the genus *Leptomias* exhibited remarkable diversity with 38 species and a total of 433 sequences, out of which *L. huangi* accounted for the highest number with 118 sequences.

The average base composition of the sequences in the DNA barcode reference library was as follows: T = 35.2%, C = 19.7%, A = 28.9%, and G = 16.2%. The content of A + T was significantly higher than that of C + G, as it is known from other arthropod taxa. The sequences exhibited a total of 273 conserved sites, 385 variable sites, 376 parsimonic information sites, and nine single mutation sites. No insertions, deletions, stop codons, or sequencing errors were detected in any of the sequences.

### 3.2 Genetic distance and species delimitation

**FIGURE 2:**
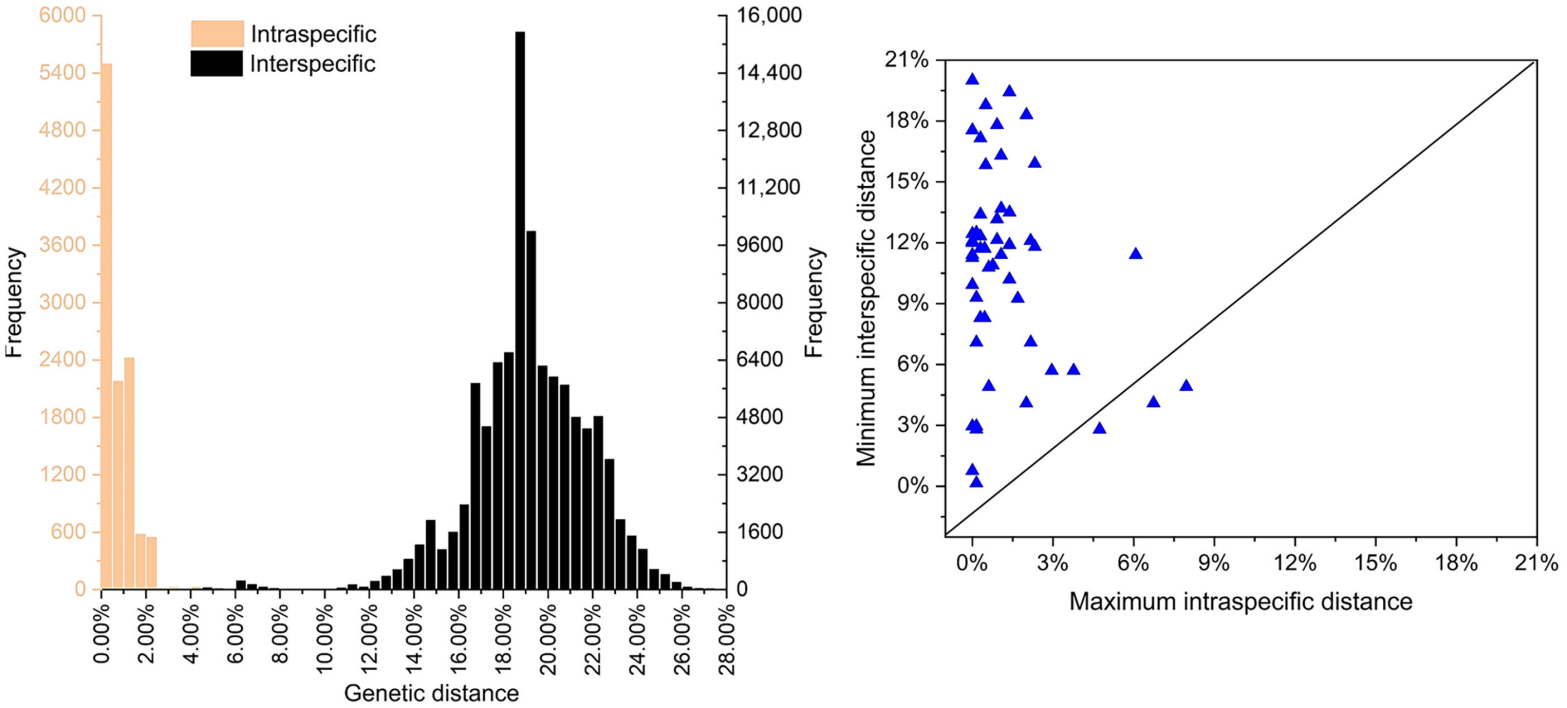
Intra‐ and inter‐GD histograms and scatter plot of the max intra‐GD versus min inter‐GD for the *Leptomias* group. Each triangle represents a species. The dot falling below the 1:1 slope indicating the absence of a barcode gap.

Our results demonstrated that the intra‐GD ranged from 0 to 7.96%, while the inter‐GD varied between 0.15% and 28.88% (Figure 2). Specifically, the intra‐GD of 41 species (77.36%) was below 2.00%, whereas the inter‐GD of 51 species (96.23%) exceeded this threshold value. Additionally, a barcode gap was observed in 51 of the total of 54 species analyzed. The highest intra‐GD value of 7.96% was found in *Leptomias midlineatus*, while *Triangulomias* sp.1RL and *T*. sp.4RL exhibited the lowest inter‐GD values at only 0.15%. On the other hand, *Pachynotus pilosus* and *L*. sp.16RL displayed the highest inter‐GD values at a staggering rate of up to 28.88%. Notably, more than half (94.3%) of species of *Leptomias* group had an intra‐GD greater than their maximum intra‐GD value observed within this study cohort as a whole. Overall, there was a substantial difference between mean inter‐ (19.15%) and intra‐ (0.76%) GDs with average values being approximately 25 times higher.

**FIGURE 3:**
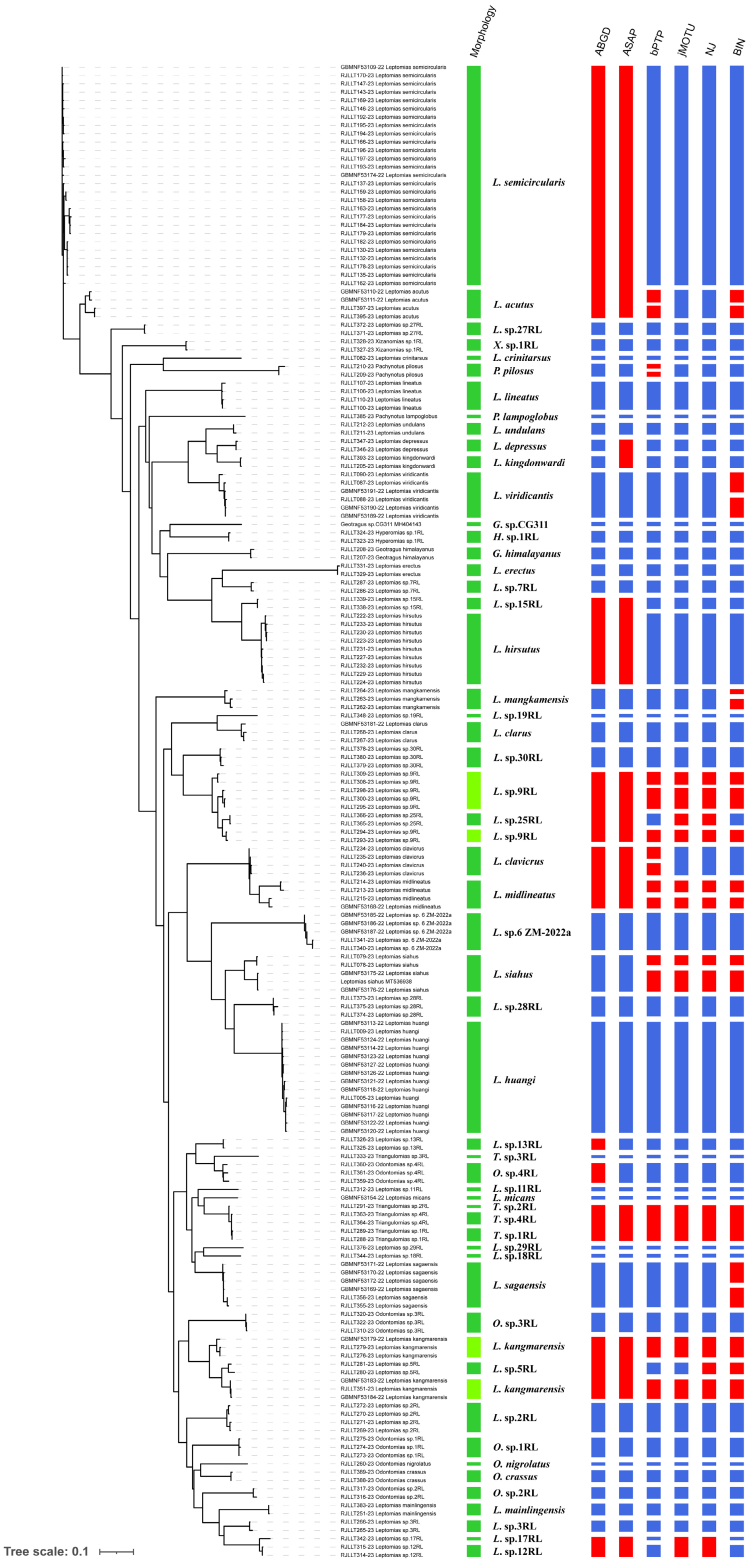
Species delimitation of *Leptomias* group based on morphology, automatic barcode gap discovery (ABGD), assemble species by automatic partitioning (ASAP), Poisson tree process (bPTP), jMOTU, neighbor‐joining (NJ) tree, and barcode index number (BIN) are shown by bars next to species names.

Based on the implementation of six methods, the number of MOTUs ranged from 45 to 63 (Figure 3). Out of the total 54 morphospecies identified based on their morphological characteristics, a significant proportion of 79.63% (43) were unambiguously discriminated by their COI sequences using various species delimitation methods. Both the ABGD and ASAP methods recognized a total of 45 MOTUs, out of which 37 corresponded with the identified morphospecies. BIN analysis yielded a result of 63 MOTUs, among which taxonomic concordance was observed for 42 MOTUs when compared to the results obtained through morphological delimitation. bPTP, jMOTU, and NJ analysis delimited a total of 61, 54, and 55 MOTUs, respectively, with respective overlaps in classification observed for approximately 43, 44, and 44 when compared to the morphological classification.

### 3.3 Investigation on taxonomic warnings

The results of our study revealed certain inconsistencies between molecular and morphological species delimitation (Table S3). Specifically, based on different species delimitation methods (Table 1), a total of four morphospecies, namely *Leptomias kangmarensis*, *L. midlineatus*, *L. siahus*, and *L*. sp.9RL were assigned to more than one MOTUs each. Furthermore, three species of *Triangulomias*, *T*. sp.1RL, *T*. sp.2RL, and *T*. sp.4RL, exhibited the lowest inter‐GD values (0–0.15%), leading to their classification as a single MOTU across six species delimitation methods (Table S3). Similarly, two species groups of *Leptomias*, *L*. sp.9RL and *L*. sp.25RL (2.80–5.05%), *L*. sp.12RL and *L*. sp.17RL (2.96%–3.12%), divided into one MOTU by four different methods.

**TABLE 1:** Four morphospecies containing possible cryptic diversity indicated by different species delimitation methods (ABGD, ASAP, bPTP, jMOTU, NJ, and BIN).

Species	The maximum intra‐GD (%)	Number of samples	Number of hap	MOTUs					
				ABGD	ASAP	bPTP	jMOTU	NJ	BIN
*L. kangmarensis*	6.74	20	6	1	1	2	2	2	3
*L. midlineatus*	7.96	8	4	1	1	2	2	2	2
*L. siahus*	6.08	7	5	1	1	2	2	2	2
*L*. sp.9RL	4.74	17	7	1	1	3	2	2	3

## 4 DISCUSSION

### 4.1 Different methods result in different species boundaries

The application of a universal threshold to all taxa is not feasible due to variations in population sizes and the timing of species differentiation (Greenstone et al., 2011). For Cryptorhynchine (Coleoptera: Curculionidae) (Astrin et al., 2012), and Entiminae (Coleoptera: Curculionidae) (Ma et al., 2022), the optimal species identification thresholds using COI are 5.00%, 3.00%, and 9.18%, respectively. In our study, accurate delimitation was achieved for 79.25% of morphospecies when the threshold of genetic distance was set at 2%. However, further scientific algorithms are required for species delimitation to avoid relying solely on empirical genetic distance thresholds (Li et al., 2023).

In the present study, six methods (ABGD, ASAP, bPTP, BIN, jMOTU, and NJ) yielded 45, 45, 61, 63, 54, and 55 MOTUs, respectively, based on a dataset of 54 morphospecies. The variations in the number of MOTUs obtained by the four species delimitation methods are likely attributed to dissimilarities in their algorithms. The BOLD database applies a generic threshold of 2.20%, with the formation of a new OTU occurring when the sample sequence threshold exceeds twice the standard (i.e., more than 4.40%) (Ma et al., 2022; Ratnasingham & Hebert, 2013). In the present study, the BIN method yielded unsatisfactory results due to the presence of a high percentage (14.8%) of species with an intra‐GD exceeding 2.2%. Consistent with previous research findings, the bPTP method frequently results in higher MOTU counts (Hsiao & Oberprieler, 2024). In addition to the input phylogenetic trees, underrepresentation of rare species or overrepresentation of species with limited intraspecies variation can impact the process of species delimitation of the bPTP method (Zhang et al., 2013). In our dataset, there is only one barcode and one haplotype available for *Geotragus* sp.CG311, while *Leptomias huangi* and *L*. semicircularis have significantly more sequences (118 and 78, respectively) and haplotypes (14 and 27, respectively) than other species. Whereas ABGD and ASAP effectively identified barcode gaps based on inter‐ and intra‐GD values to segment the data (Puillandre et al., 2012, 2021). In line with prior research, these two approaches produced relatively conservative outcomes (Hsiao & Oberprieler, 2024; Li et al., 2023; Song et al., 2018). Higher intra‐GD such as *L. siahus* (6.08%) and lower inter‐GD such as *L*. sp.9RL and *L*. sp.25RL (2.80%–5.05%) affect the ability of these two methods to define species. The jMOTU method was specifically designed to optimize global comparison using the Needleman–Wunsch (NW) algorithm and generate OTUs based on user‐selected cut‐off values (Jones et al., 2011). The analytical package jMOTU yielded 54 MOTUs when employing single clustering and a selected cut‐off of 22 (3.34%). There is no standardized algorithm or input parameter that can optimally restore the true species boundary for all organisms (Ratnasingham & Hebert, 2013). Especially when dealing with large‐scale datasets, it becomes imperative to adopt an integrated approach to effectively classify species (Li et al., 2023; Song et al., 2018).

### 4.2 Geographical population diversity versus cryptic species diversity

**FIGURE 4:**
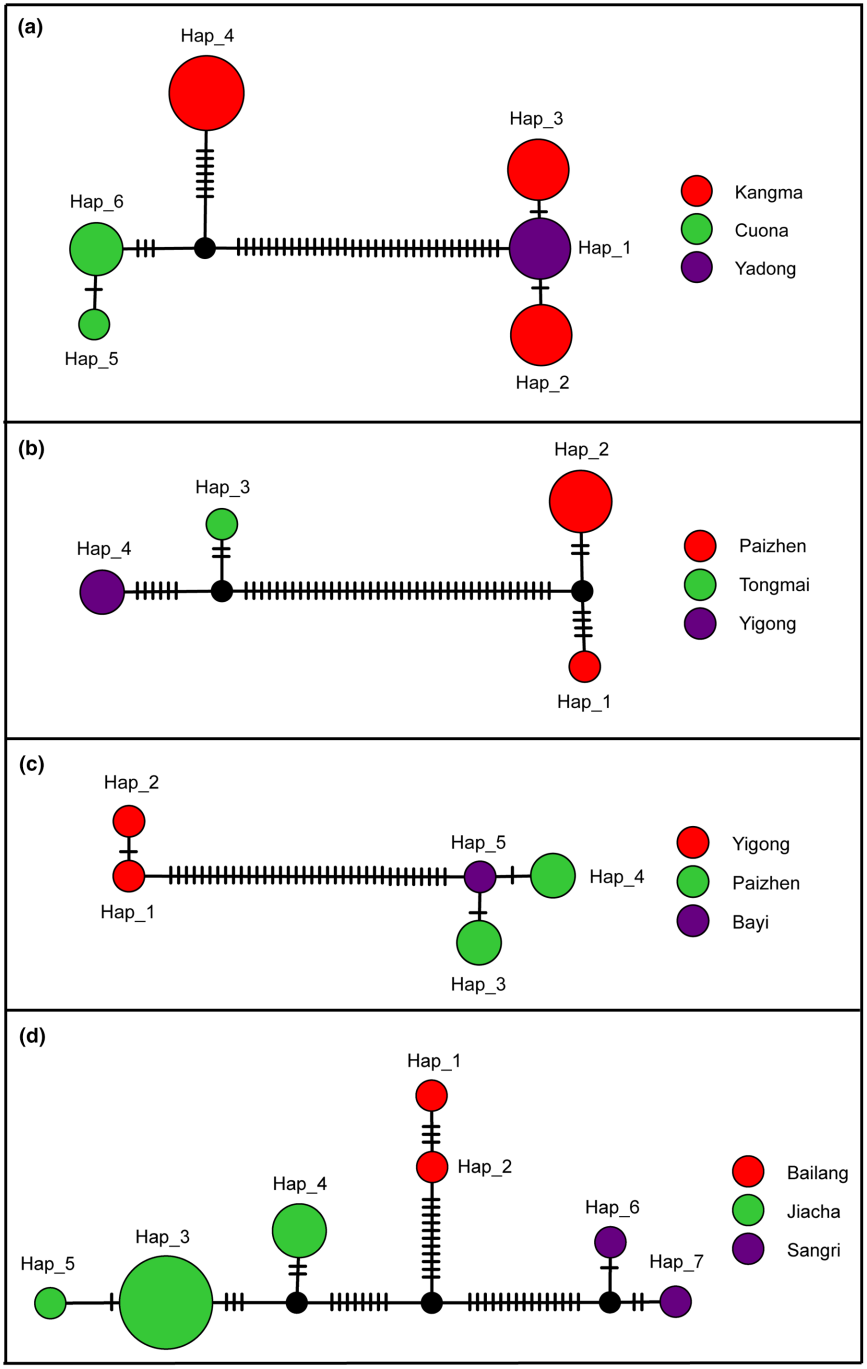
Haplotype networks for species with multiple MOTUs based on COI sequences. (a) *Leptomias kangmarensis*; (b) *L*. *midlineatus*; (c) *L. siahus*, and (d) *L*. sp.9RL. The circles represent different haplotypes, and the short line segments indicate mutated positions between haplotypes. Different colors and sizes of the circles represent geographical regions and relative numbers of sequences.

Due to hind wing degeneration, *Leptomias* generic group species are incapable of flight. They exhibit a higher likelihood of accumulating genetic variation due to geographic isolation, which may suggest the presence of cryptic species without distinct morphological divergence. Haplotype network analyses revealed that four species were segregated into multiple MOTUs based on distinct geographical distributions. The long‐term geographic isolation of these species may drive the divergence of distinct lineages. For instance, *Leptomias kangmarensis*, with higher intra‐GD (0–6.74%), exhibited three BINs (ADA5851, AET7873, and AFG9943) and two MOTUs in bPTP, jMOTU, and NJ analyses (Table 1). Among the sequences of *L*. *kangmarensis*, three sequences from Jilong, three sequences from Yadong, and three sequences from Rikaze. The haplotype network analysis effectively demonstrated geographical populations (Figure 4a). Consequently, DNA barcodes may revealed cryptic species within the *L*. *kangmarensis* species complex. Several similar cases were also illustrated. *L*. *midlineatus* (Table 1; Figure 4b), *L*. *siahus* (Table 1; Figure 4c), and *L* sp.9RL (Table 1; Figure 4d) exhibited multiple MOTUs in BIN, bPTP, jMOTU, and NJ analyses. Additionally, the haplotype network analysis revealed potential cryptic species within these complexes.

**FIGURE 5:**
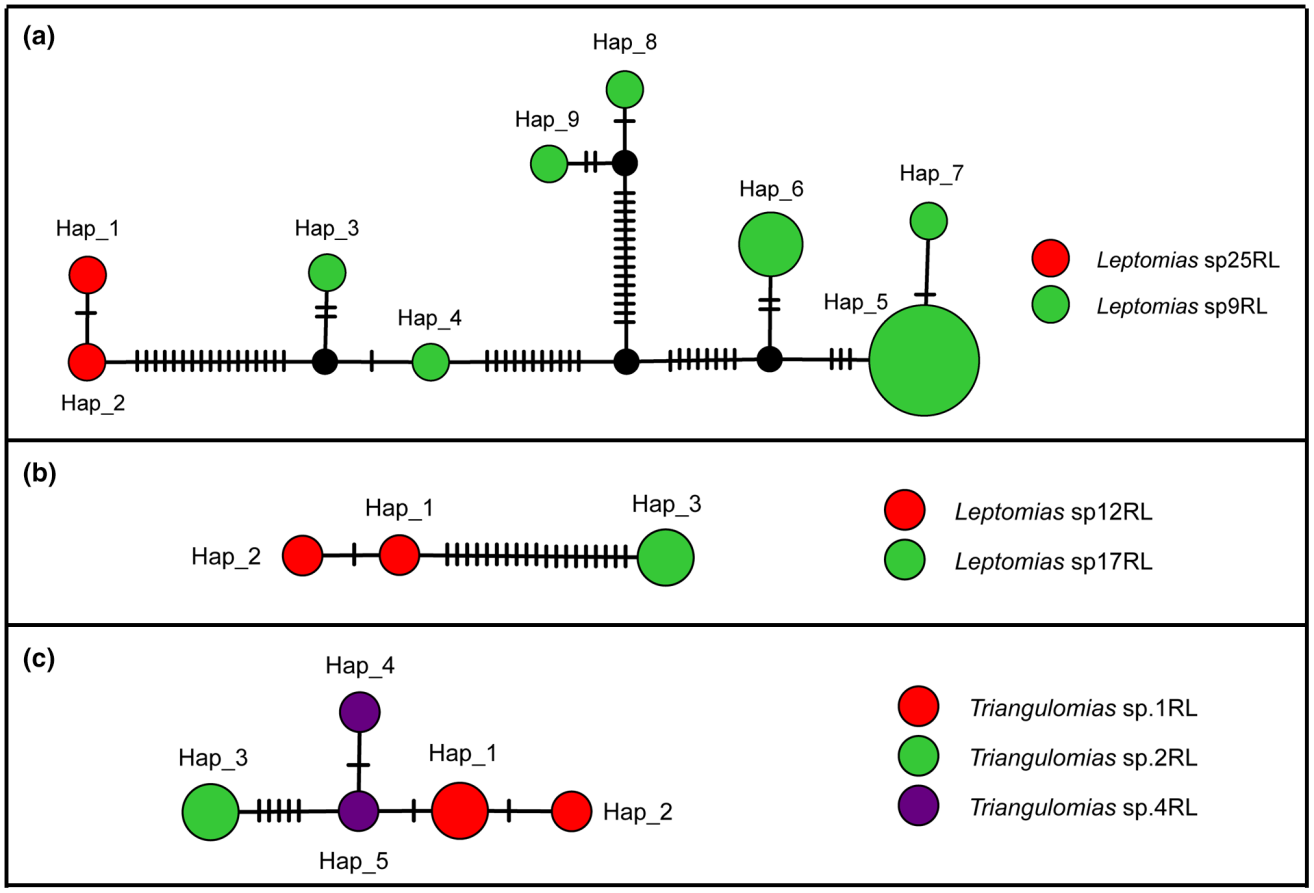
Haplotype networks for species groups divided into one MOTU by multiple delimitation methods based on COI sequences. The circles represent different haplotypes, and the short line segments indicate mutated positions between haplotypes. Different colors and sizes of the circles represent different species and relative numbers of sequences.

In our dataset, two *Leptomias* species groups, *Leptomias* sp.12RL and *L*. sp.17RL, *L*. sp.9RL, and *L*. sp.25RL, with lower inter‐GD (2.96%–3.12% and 2.80%–5.05%) assigned to the same MOTU by ABGD, ASAP, jMOTU, and NJ. Haplotype network analysis did not reveal the presence of shared haplotypes (Figure 5a,b). And we found significant differences in the male genitalia of these species after dissection. Meanwhile, we observed that *Triangulomias* sp.1RL, *T*. sp.2RL, and *T*. sp.4RL were consistently assigned to the same MOTU using different species delimitation methods (Table S3). Additionally, haplotype network analysis revealed the absence of shared haplotypes among these three morphospecies (Figure 5c). The species *T*. sp.1RL, *T*. sp.2RL, and *T*. sp.4RL were found to have three, two, and two sequences, respectively, with inter‐GD ranging from 0.15% to 0.77%. We generated the sequences of these three species ourselves, and they were collected in counties Dingjie (2491 m), Milin (2943 m), and Dingri (4294 m). We conducted a re‐examination of the voucher specimens for several species. After careful examination, we observed distinct variations among these three species. Dorsal surface of rostrum with a narrow and shallow median sulcus, exceed fore margin of forehead; scutellum distinct, U‐shaped; interstriae of elytra with 3–4 rows of setae; fore tibiae slightly projected outwards, with several extremely small teeth in *T*. sp.1RL. Dorsal surface of rostrum with a narrow and shallow median sulcus, reaching fore margin of forehead; scutellum distinct, U‐shaped; interstriae of elytra with 3–4 rows of setae; fore tibiae obviously projected outwards, with several distinct teeth in *T*. sp.2RL. Dorsal surface of rostrum with a narrow and shallow median sulcus, reaching fore margin of forehead; scutellum not obvious; interstriae of elytra with 2–3 rows of setae; fore tibiae slightly projected outwards, without teeth in *T*. sp.4RL. Due to the lack of corresponding specimens, we were unable to provide a description of male genitalia. These cases demonstrate that in some taxa, it is difficult to accurately define species solely through external morphology or a small number of molecular markers. In the future, we should combine morphological, molecular, and ecological evidence to avoid errors caused by a single method.

### 4.3 Revision of the current taxonomic classification

Through the comparison of genetic sequences and morphological examination of voucher specimens, we have successfully utilized DNA barcode technology to rapidly identify and revise the current taxonomic classifications within the species of *Leptomias* group (Table S4). For instance, by comparing sequences, we were able to identify *Leptomias* sp.1RL as *L. clarus*. We re‐examined the voucher specimens of *L*. sp.1RL and *L. clarus*, and found that several features can serve as evidence: A laterally positioned triangular depression on the rostrum between the eyes and antennal scrobes in dorsal view; prementum with six setae; antennae scape reaching the fore margin of eyes at rest, among others. Most importantly, there is no difference in their male genitalia. Similarly, both *Odontomias latus* and *O*. sp.5RL were identified as *O. nigrolatus* based on these characteristics: A laterally positioned deep sulcus on the rostrum between the eyes and antennal scrobes; U‐shaped scutellum; small teeth present on fore‐ and median tibiae; interstriae with 2–3 column setae.

### 4.4 Limitations of single gene

The combination of multiple morphospecies into a single MOTU, or the division of a morphospecies into multiple MOTUs, highlights the potential limitations of using mitochondrial single genes for species identification. The molecular indistinguishability of some species may be attributed to the recent divergence of these groups, resulting in insufficient accumulation of molecular differences (Dupuis et al., 2012; Knowles & Carstens, 2007; Maddison, 1997). Additionally, in the case of Wolbachia infection, the use of a single mtDNA marker may hinder the interspecific demarcation of some taxa. Because mtDNA‐based phylogenetic and nucleotide differences in these populations increase or decrease according to the timing and number of intrusions by endosymbionts, this may lead to divergence in evolutionary history in terms of mitochondria, nuclear genes, and morphology (Hurst & Jiggins, 2005; Whitworth et al., 2007). Further, the results of species identification are also potentially impacted by other factors including mitochondrial DNA recombination revisited (Rokas et al., 2003), the presence of numts (Song et al., 2008), incomplete lineage sorting (Nabholz, 2024), and insufficient sampling of taxon (Bergsten et al., 2012; Meyer & Paulay, 2005). Therefore, integrated taxonomy combining molecular, morphological, geographic, and ecological data is crucial for exploring species boundaries (de Queiroz, 2007; Ross et al., 2010).

## 5 CONCLUSION

Overall, DNA barcodes can successfully delimit species of *Leptomias* group, with a matching rate of 79.6% with morphological species. By comparing the performance of different analysis tools, methods of jMOTU fit well with the *Leptomias* group COI barcode dataset. Unusual deep intraspecific differentiation was detected in some species complexes, indicating potential morphological misidentification or cryptic species. There is an urgent need to integrate more nuclear genes, morphological characteristics of different life stages, and ecological data for further research.

## AUTHOR CONTRIBUTIONS

**Jinliang Ren:** Conceptualization (lead); data curation (lead); formal analysis (lead); investigation (lead); methodology (lead); project administration (lead); resources (lead); software (lead); validation (lead); visualization (lead); writing – original draft (lead). **Li Ren:** Conceptualization (supporting). **Runzhi Zhang:** Conceptualization (supporting); funding acquisition (supporting); writing – review and editing (equal).

## FUNDING INFORMATION

This research was funded by the National Natural Science Foundation of China (31872260).

## CONFLICT OF INTEREST STATEMENT

The authors declare no conflict of interest.

## Supporting information


Tables S1‐S4

## Data Availability

The data presented in this study are available in Supplementary Material.
